# Phenotypic Expression of ADAMTS13 in Glomerular Endothelial Cells

**DOI:** 10.1371/journal.pone.0021587

**Published:** 2011-06-24

**Authors:** Ramesh Tati, Ann-Charlotte Kristoffersson, Anne-lie Ståhl, Matthias Mörgelin, David Motto, Simon Satchell, Peter Mathieson, Minola Manea-Hedström, Diana Karpman

**Affiliations:** 1 Department of Pediatrics, Clinical Sciences Lund, Lund University, Lund, Sweden; 2 Division of Clinical and Experimental Infection Medicine, Clinical Sciences Lund, Lund University, Lund, Sweden; 3 Department of Internal Medicine and Pediatrics, University of Iowa College of Medicine, Iowa City, Iowa, United States of America; 4 Academic Renal Unit, University of Bristol, Southmead Hospital, Bristol, United Kingdom; Universidade de Sao Paulo, Brazil

## Abstract

**Background:**

ADAMTS13 is the physiological von Willebrand factor (VWF)-cleaving protease. The aim of this study was to examine ADAMTS13 expression in kidneys from ADAMTS13 wild-type (*Adamts13^+/+^*) and deficient (*Adamts13^−/−^*) mice and to investigate the expression pattern and bioactivity in human glomerular endothelial cells.

**Methodology/Principal Findings:**

Immunohistochemistry was performed on kidney sections from ADAMTS13 wild-type and ADAMTS13-deficient mice. Phenotypic differences were examined by ultramorphology. ADAMTS13 expression in human glomerular endothelial cells and dermal microvascular endothelial cells was investigated by real-time PCR, flow cytometry, immunofluorescence and immunoblotting. VWF cleavage was demonstrated by multimer structure analysis and immunoblotting. ADAMTS13 was demonstrated in glomerular endothelial cells in *Adamts13^+/+^* mice but no staining was visible in tissue from *Adamts13^−/−^* mice. Thickening of glomerular capillaries with platelet deposition on the vessel wall was detected in *Adamts13^−/−^* mice. ADAMTS13 mRNA and protein were detected in both human endothelial cells and the protease was secreted. ADAMTS13 activity was demonstrated in glomerular endothelial cells as cleavage of VWF.

**Conclusions/Significance:**

Glomerular endothelial cells express and secrete ADAMTS13. The proteolytic activity could have a protective effect preventing deposition of platelets along capillary lumina under the conditions of high shear stress present in glomerular capillaries.

## Introduction

The known substrate for ADAMTS13 (a disintegrin-like and metalloprotease with thrombospondin type-1 motifs) is von Willebrand Factor (VWF) [1], a glycoprotein, that induces platelet adhesion and aggregation at sites of vascular injury and high-shear stress [2]. VWF is produced in endothelial cells [3] and megakaryocytes [4] and secreted from endothelial cells as ultra-large multimers (ULVWF) [5], which are biologically very active [2], [6].

ULVWF multimers are cleaved on the surface of endothelial cells into smaller multimers by ADAMTS13 [7]. ADAMTS13 cleaves the 1605Tyr-1606Met peptide bond in the A2 domain of VWF thereby releasing 140 kDa and 176 kDa VWF fragments [8]. Apart from ADAMTS13, four other proteases, elastase, proteinase 3, cathepsin G and matrix metalloprotease 9 (MMP9), have been shown to cleave VWF at sites identical with, or near, the ADAMTS13 cleavage site [9]. ADAMTS13 is, however, considered most important for cleavage of VWF under physiological conditions and conditions of increased shear stress [7].

Deficient ADAMTS13 activity leads to thrombotic thrombocytopenic purpura (TTP) [10] which may either be the result of mutations in the ADAMTS13 gene (congenital TTP) [11] or due to the presence of auto-antibodies against ADAMTS13 (acquired TTP) [12]. TTP is characterized by thrombocytopenia, microangiopathic hemolytic anemia, fever, renal and neurological manifestations. Due to lack of or dysfunction of ADAMTS13, the degradation of ULVWF is impaired which leads to the formation of disseminated platelet thrombi, a characteristic feature of TTP [10].

ADAMTS13 has been found to be synthesized by hepatic stellate cells [13], endothelial cells [14], [15] and megakaryocytes [16], [17], as well as other cells. The kidney has been shown to express ADAMTS13 mRNA [11], [18]. As the kidney is one of the main organs affected during TTP, our group has studied renal expression of ADAMTS13. ADAMTS13 was demonstrated *in situ* in the renal cortex [19]. ADAMTS13 expression was detected at both the mRNA and protein level in cultured podocytes and tubular cells and its bioactivity was demonstrated in both cell types [19], [20].

ADAMTS13 cleaves ULVWF multimers on the surface of endothelial cells under flow conditions mimicking the bloodstream [7]. This form of cleavage would be of utmost importance in the presence of high shear stress such as in glomerular capillaries. Deficient ADAMTS13 or dysfunctional protease activity would presumably allow deposition of ULVWF and platelets on glomerular capillary walls contributing to the development of thrombotic microangiopathy. ADAMTS13-deficient mice (with the 129X1/SvJ and C57BL/6J genetic background) did not develop TTP-like pathology spontaneously but, upon introduction of the CASA/Rk background, were shown to develop TTP-like pathology after endothelial cell injury was induced by Shiga toxin [21].

The purpose of the present study was to investigate glomerular endothelial ADAMTS13 expression and phenotype using renal tissue from wild-type and ADAMTS13-deficient mice and to study the effect of ADAMTS13 deficiency on glomerular capillary walls and platelet deposition. Furthermore, in vitro studies were designed to demonstrate ADAMTS13 expression and activity in human glomerular endothelial cells.

## Results

### ADAMTS13 expression in mouse kidney

**Figure 1:**
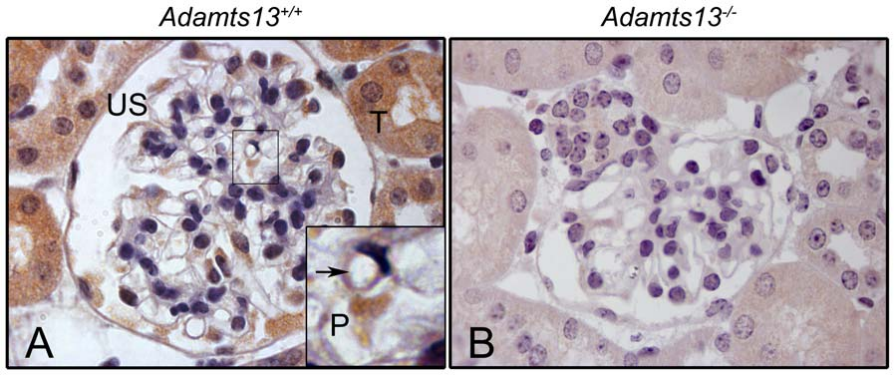
ADAMTS13 expression in mouse kidney. ADAMTS13 expression was investigated by immunohistochemistry in mouse renal tissue. Staining was observed in the kidney of *Adamts13^+/+^* wild-type mice on the mixed 129X1/SvJ and C57BL/6 genetic background (Panel A). The inset in panel A shows glomerular capillary staining (arrow) at a higher magnification. Tissue from *Adamts13^−/−^* mice (same genetic background) did not label for ADAMTS13 (Panel B). Reproducible results were obtained in six separate experiments including mice with the mixed C57BL/6J and CAST/Ei genetic background. Images are at 1000x magnification. P: podocyte, US: urinary space, T: tubular cell.

Immunohistochemistry performed on renal tissue from *Adamts13^+/+^* wild-type mice exhibited positive staining in glomerular endothelial cells (Figure 1A) as well as in podocytes and tubuli. No staining was visible in tissue from the *Adamts13^−/−^* mice (Figure 1B). The control antibodies did not label mouse tissue (data not shown). No signal was detected when the primary antibodies were omitted (data not shown).

### Altered vessel phenotype in ADAMTS13-deficient mice

**Figure 2:**
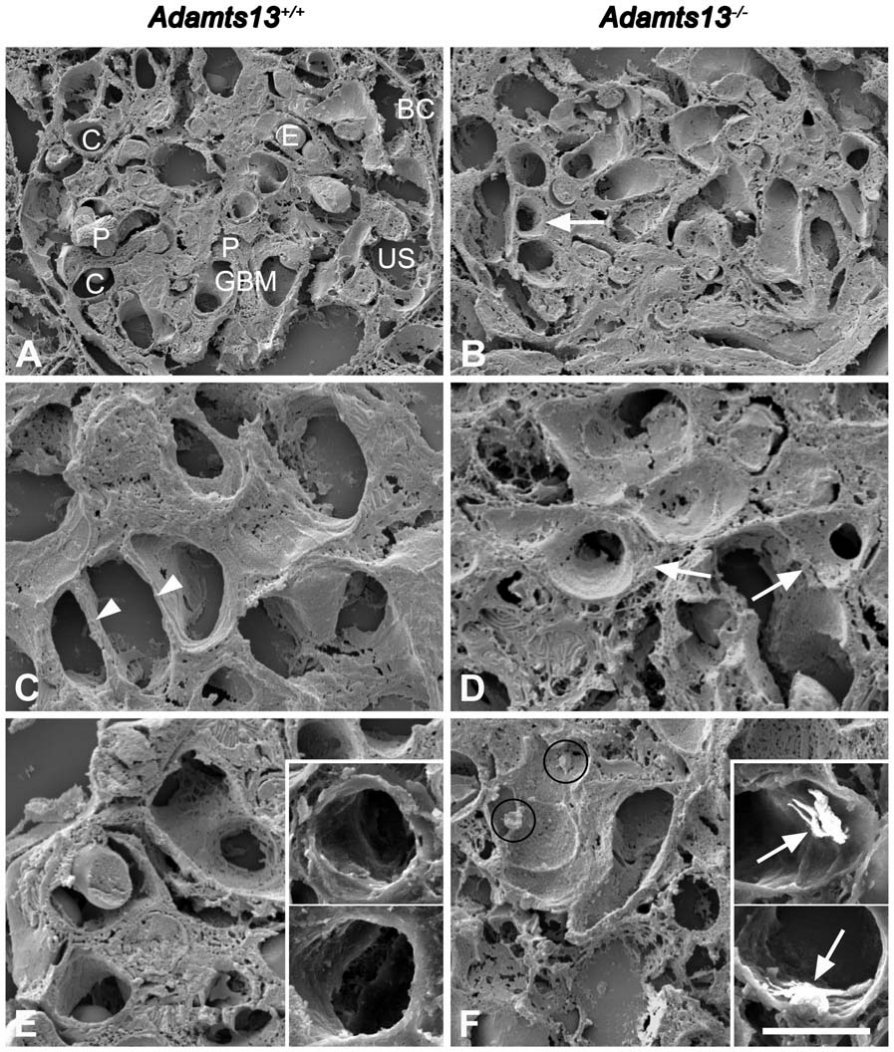
Ultramorphology of glomeruli in wild-type and deficient mice. Scanning electron microscopy of kidney from wild-type mice showed normal morphology as depicted in panel A. In the renal glomeruli the Bowman's capsule (BC), glomerular basement membrane (GBM), endothelial cell capillaries (C), erythrocytes (E), podocytes (P) and urinary space (US) were identified. Panel B shows a glomerulus from an *Adamts13^−/−^* mouse demonstrating thickened and irregular capillary walls (arrow). These findings were enlarged in panel C from another glomerulus from an *Adamts13^+/+^* mouse showing thin glomerular capillary walls (arrow heads) and in panel D from an *Adamts13^−/−^* mouse showing thickened capillary walls (arrows) with deposits on the vessel lumina. Panel E demonstrates lack of platelet deposits in glomerular capillaries from an *Adamts13^+/+^*mouse, further confirmed by immunoelectron microscopy (insets). Panel F was taken from another glomerulus in an *Adamts13^−/−^* mouse and shows platelets (within circles) deposited on a vessel wall. Insets in panel F show immunoelectron microscopy with labeled platelets (arrows) deposited on glomerular capillary walls. Scale bar represents 10 µm (A, B), 5 µm (C–F) and 2.5 µm (insets in E, F). Panels A, B, D–F and insets were from mice with the 129X1/SvJ and C57BL/6 genetic background and panel C was from mice with the C57BL/6J and CAST/Ei genetic background.

In order to assess if lack of ADAMTS13 affected the vessel wall, renal samples from *Adamts13^+/+^* (Figure 2A, C, E) and *Adamts13^−/−^* (Figure 2B, D, F) mice (from two independent genetic backgrounds) were examined by scanning electron microscopy. Glomeruli from *Adamts13^+/+^* wild-type mice exhibited patent capillaries with smooth vessel walls and thin basement membranes as shown in Figure 2A and 2C. Glomeruli from *Adamts13^−/−^* mice exhibited thickened and irregular vessel walls (Figure 2B and 2D). This method could not differentiate between intimal proliferation and thickening of the glomerular basement membrane both of which could explain this finding. Uneven surfaces inside the vessel wall suggested protein deposition. In comparison to *Adamts13^+/+^* mice, *Adamts13^−/−^* mice exhibited more platelet deposits in glomerular capillaries (Figures 2E and 2F, respectively) suggesting that lack of ADAMTS13 contributed to platelet binding to the capillary walls. To quantify platelet deposition in the glomerular capillaries immunoelectron microscopy was performed using an antibody against the platelet marker integrin-β3. Platelet deposition appeared as bright labeling (insets in Figure 2F). The control antibody showed no staining (data not shown).

Vessel wall thickness and platelet deposition on glomerular capillary walls were quantified and compared between *Adamts13^+/+^* and *Adamts13^−/−^* mice showing that *Adamts13^−/−^* mice had significantly thicker capillary walls and more platelet deposits, as presented in Table 1.

**Table 1:** Vessel wall thickness and platelet deposition in glomerular capillaries of *Adamts13^+/+^* and *Adamts13^−/−^* mice.

	*Adamts13^+/+^* mice (n = 5)	*Adamts13^−/−^* mice (n = 5)	*P* value
Capillary wall thickness (µm)a, b	0.5 (0.3−0.8)	1.2 (0.2−2.3)	0.005^d^
Platelet deposits/mm^2^ a, c	51 (41−59)	890 (800−981)	0.007^d^

a median and (range). ^b^Assessed by quantifying capillary walls in 30 glomerular profiles from each mouse. ^c^Assessed by counting the number of labeled platelets in all visible vessels in 30 glomerular profiles from each mouse. ^d^For each mouse a median value was obtained. Medians of five mice in each group were compared statistically.

### ADAMTS13 mRNA expression in endothelial cells

**Figure 3:**
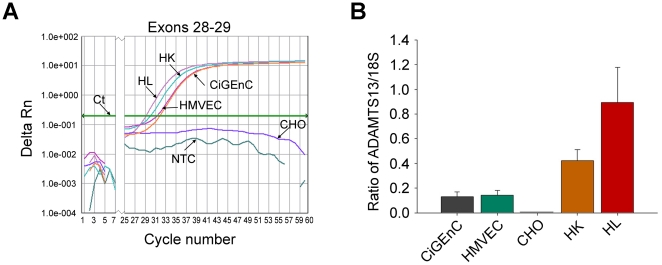
ADAMTS13 mRNA expression in cultured endothelial cells. *ADAMTS13* gene transcripts in endothelial cells were detected by real-time PCR. A. Using the probe against exons 28–29 the amplification plot indicates detectable *ADAMTS13* mRNA levels in CiGEnC and HMVEC cells. Human kidney (HK) and human liver (HL) were used as the positive controls. No template control (NTC) and CHO cells were used as the negative controls and showed no amplification. B. The housekeeping gene 18S, which was expressed at comparable levels in all samples, was used to standardize the data. The upper limit of the boxes depicts the means and bars are standard error of the mean from three separate cell culture experiments.

Real-time PCR was applied to investigate if glomerular endothelial cells CiGEnC synthesize ADAMTS13. HMVEC were studied as an endothelial cell control. *ADAMTS13* mRNA was detected in CiGEnC and HMVEC (Figure 3) but not in CHO cells used as the negative control. Human kidney pool and human liver, which have previously been shown to express *ADAMTS13* mRNA [20] were used as positive controls. No-template control did not show any amplification (Figure 3A). 18S rRNA was used as an endogenous control and showed comparable levels of expression in all samples. Results in Figure 3B are presented as the ratio of ADAMTS13/18S.

### ADAMTS13 expression in cultured endothelial cells

**Figure 4:**
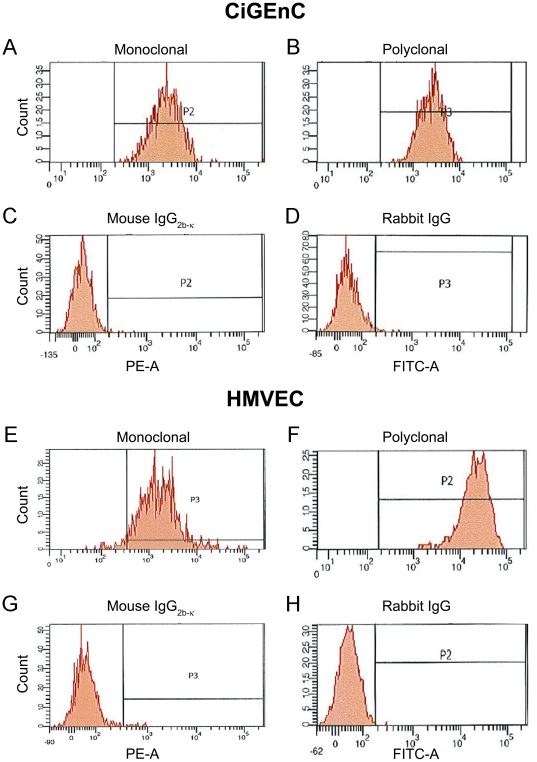
Detection ofADAMTS13 in cultured endothelial cells by flow cytometry. ADAMTS13 protein was detected in CiGEnC (Panels A–D) using A10 monoclonal antibody (panel A) and SU19 polyclonal antibody (panel B). The control antibodies, mouse IgG_2b-κ_ (panel C) and rabbit IgG (panel D) did not bind to any cells. HMVEC showed similar results (Panels E–H). Reproducible results were obtained from two different experiments.

ADAMTS13 was detected in both CiGEnC and HMVEC by flow cytometry analysis of cells permeabilized with Triton X-100. In CiGEnC both the monoclonal anti-ADAMTS13 A10 antibody and the polyclonal SU19 antibody bound to 99% of the population indicating that the entire population was positive for ADAMTS13 expression (Figure 4A and 4B, respectively). No labeling was demonstrated using the control antibodies (Figure 4C and 4D). Similar results were demonstrated for the HMVEC cells (Figures 4E–4H) and even here most of the labeling was intracellular. No signal was detected when the primary antibodies were omitted (data not shown).

**Figure 5:**
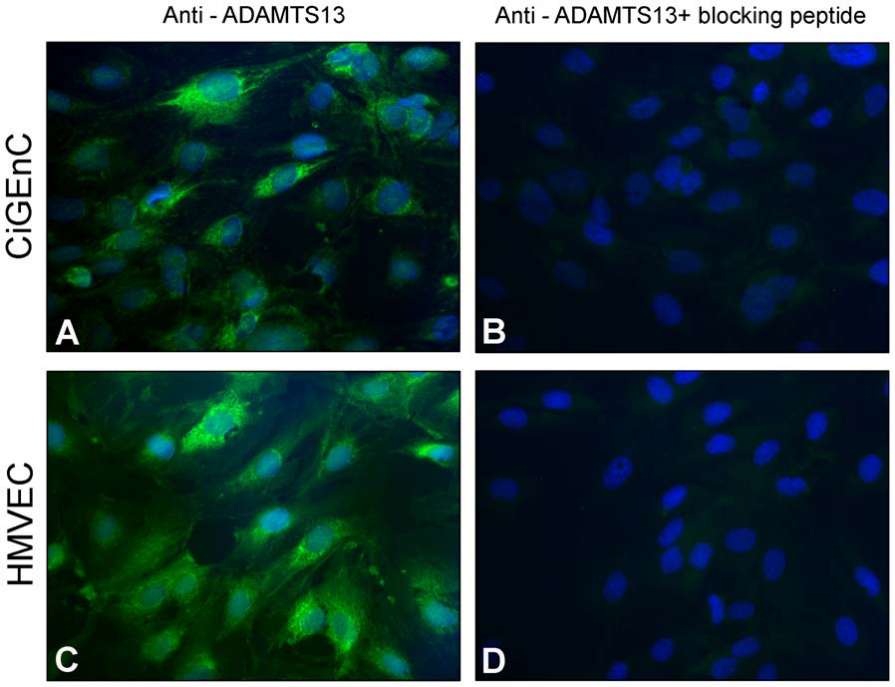
ADAMTS13 expression detected intracellularly by immunofluorescence. ADAMTS13 protein expression was investigated in CiGEnC (panels A–B) and HMVEC (panels C–D). ADAMTS13 was detected in CiGEnC using SU19 polyclonal antibody (panel A). The SU19 antibody was pre-incubated with blocking peptide resulting in marked signal reduction (panel B) in which FITC labeling of ADAMTS13 was abolished and blue DAPI labeling marked cell nuclei. HMVEC cells showed similar results staining positively for ADAMTS13 with the polyclonal SU19 antibody (C). SU19 blocking experiments resulted in marked decrease in signal intensity (D). Reproducible results were achieved in at least six separate cell experiments from three different passages. All images are at 400x magnification**.**

ADAMTS13 expression in endothelial cells was further investigated by immunofluorescence. ADAMTS13 protein was detected intracellularly in permeabilized CiGEnC using the monoclonal and the polyclonal antibodies. Results using the polyclonal anti-ADAMTS13 antibody are presented in Figure 5A. Pre-incubation with blocking peptide markedly reduced signal intensity of the polyclonal antibody (Figure 5B). Similar results were observed with the HMVEC cells (Figures 5C–5D). Comparable results were obtained using the monoclonal anti-ADAMTS13 antibody (data not shown). Omission of the primary antibodies resulted in lack of signal (data not shown).

**Figure 6:**
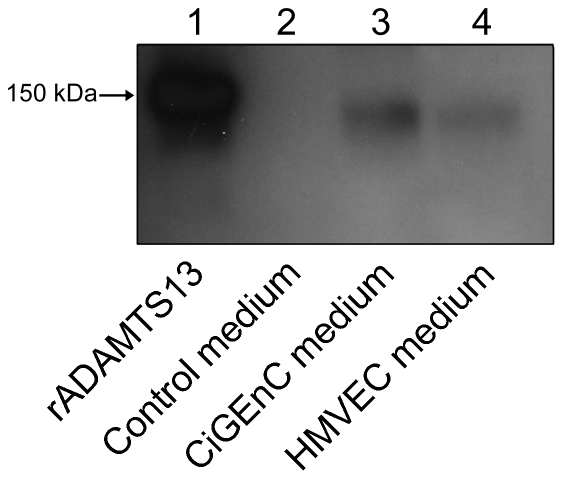
ADAMTS13 is secreted into the media. Recombinant ADAMTS13 showed a band at 150 kDa under non-reducing conditions (lane 1, the positive control) and control medium did not show any band (lane 2). Media from CiGEnC and HMVEC exhibited similar bands at approximately 150 kDa (lanes 3 and 4, respectively). Reproducible results were obtained from four different experiments.

ADAMTS13 in the media from both endothelial cells was detected by immunoblotting. Purified recombinant human ADAMTS13 was used as the positive control showing a band at 150 kDa (Figure 6, lane 1). A band was not detected in the control media (Figure 6, lane 2). Media from both CiGEnC and HMVEC cells revealed bands at approximately 150 kDa (Figure 6, lanes 3 and 4, respectively).

### ADAMTS13 activity in endothelial cells

**Figure 7:**
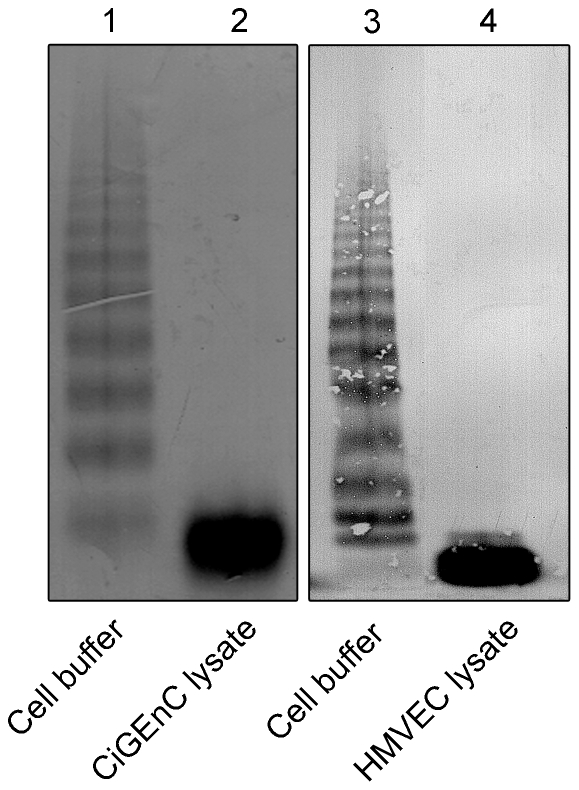
ADAMTS13 activity depicted by the VWF multimer structure. Endothelial cell lysates were incubated with purified VWF. Cell buffer incubated with VWF was used as the negative control and showed high molecular weight VWF multimers (lanes 1 and 3). CiGEnC and HMVEC lysates showed marked cleavage of VWF multimers (lanes 2 and 4, respectively). The two panels were run on separate gels but within each panel samples were run on the same gel. Reproducible results were obtained from three separate experiments.

The VWF cleavage activity of ADAMTS13 in both endothelial cells was demonstrated by VWF multimer structure analysis. Cell buffer did not exhibit any cleavage and exogenously added VWF exhibited typical multimers (Figure 7, lanes 1 and 3). In the presence of CiGEnC and HMVEC lysates almost complete breakdown of VWF multimers was demonstrated (Figure 7, lanes 2 and 4, respectively).

### Cleavage specificity of ADAMTS13

**Figure 8:**
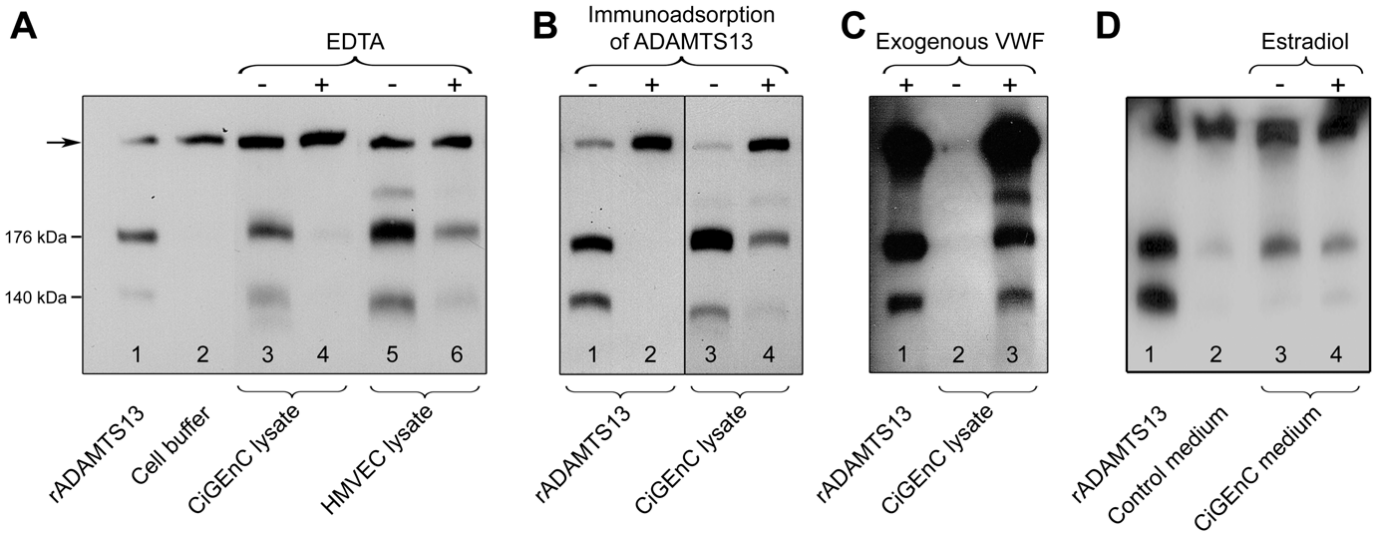
VWF cleavage by endothelial cell lysates depicted by immunoblotting. A. Recombinant ADAMTS13 cleaves the A2 domain of VWF whereby 176 kDa and 140 kDa bands appear (lane 1). Full-length VWF is depicted by an arrow. Cell buffer, incubated with VWF and used as the negative control, did not exhibit the cleavage fragments (lane 2). CiGEnC cell lysate exhibited VWF cleaving activity as shown by the presence of similar cleavage fragments (lane 3) which were inhibited by 20 mM EDTA (lane 4). HMVEC lysate also showed the two breakdown products (lane 5) which were partially inhibited by EDTA (lane 6). B. Immunoadsorption of ADAMTS13 inhibited VWF cleaving activity. Recombinant ADAMTS13 showed the two cleavage fragments (lane 1) and the cleaving activity was abrogated by removal of ADAMTS13 (lane 2). CiGEnC cell lysate also induced VWF cleavage (lane 3) which was inhibited by immunoadsorption of ADAMTS13 from the lysate (lane 4). Experiments were carried out three times with reproducible results. C. VWF cleavage products were derived from exogenous VWF. Exogenous VWF was added to rADAMTS13 resulting in two cleavage products (lane 1). CiGEnC lysate, without added exogenous VWF, did not show cleavage bands but exhibited a weak band corresponding to full-length VWF (lane 2). When purified exogenous VWF was added to CiGEnC lysates VWF cleavage was demonstrated (lane 3). D. Recombinant ADAMTS13 cleaves exogenously added VWF in cell medium (that was not in contact with endothelial cells, lane 1). Cell medium (that had not been exposed to cells) without added ADAMTS13 exhibited full-length VWF and a very weak band at 176 kDa (lane 2). Cell medium derived from unstimulated CiGEnC exhibited a stronger band at 176 kDa and a weak band at 140 kDa (lane 3). Cell medium derived from CiGEnC stimulated with estradiol exhibited a band at 176 kDa and a weak band at 140 kDa (lane 4). All lanes were run on the same gel. Experiments were carried out twice with reproducible results.

ADAMTS13 cleaves VWF in the A2 domain at the 1605Tyr-1606Met peptide bond thereby releasing 140 kDa and 176 kDa fragments [8]. To investigate cleavage specificity, cell lysates and media were incubated with or without VWF and immunoblotting was carried out using anti-VWF antibodies specific for the cleavage products. Recombinant ADAMTS13 incubated with VWF (as the positive control) cleaved VWF as expected to 176 kDa and 140 kDa fragments (Figure 8A lane 1) whereas cell buffer incubated with VWF showed only the full-length VWF (Figure 8A, lane 2). CiGEnC cell lysate exhibited cleavage bands (Figure 8A, lane 3) similar to those induced by rADAMTS13. This cleavage was inhibited by pre-incubation with EDTA suggesting that the cleaving enzyme was a metalloprotease (Figure 8A, lane 4). Similar results were obtained with HMVEC cell lysates showing the two cleaved VWF fragments (Figure 8A, lane 5) and marked inhibition by pre-incubation with EDTA (Figure 8A, lane 6).

As the protease inhibitor used could inhibit all proteases, except metalloproteases, the data presented above indicated that the endothelial cell lysates contained a metalloprotease capable of cleaving VWF. To show that the cleaving activity could be specifically ascribed to ADAMTS13 the lysates were immunoadsorbed using anti-ADAMTS13 antibody. As a positive control rADAMTS13 showed cleavage of VWF (Figure 8B lane 1) which was inhibited by immunoadsorption of ADAMTS13 (Figure 8B, lane 2). Similarly, the lysate from CiGEnC exhibited VWF cleaving activity (Figure 8B, lane 3) which was markedly reduced by immunoadsorption of ADAMTS13 (Figure 8B, lane 4). Similar results were obtained using HMVEC cell lysates (data not shown).

CiGEnC lysate, to which exogenous VWF was not added, exhibited a weak band at approximately 270 kDa corresponding to full-length VWF (Figure 8C lane 2) but did not exhibit VWF cleavage products. Cell media, to which exogenous VWF was not added, also showed a weak band corresponding to full-length VWF and lack of VWF cleavage activity (data not shown). Similar results were obtained using the lysates and media of HMVEC without addition of exogenous VWF (data not shown). Thus the demonstrated VWF cleavage in cell lysates occurred in exogenously added VWF.

Media from CiGEnC incubated with VWF exhibited minimal cleavage of VWF as visualized by the appearance of the 140 kDa and 176 kDa fragments (Figure 8D lane 3). CiGEnC were stimulated with PMA, histamine or estradiol. Media taken from these stimulated cells also exhibited weak bands at 140 kDa and 176 kDa (Figure 8D, lane 4 shows media from cells stimulated with estradiol), albeit not more than media from unstimulated cells. These experiments were not carried out using media from HMVEC.

Taken together, these results indicate that active ADAMTS13 is present inside glomerular endothelial cells and a small amount is secreted into the cell media but the activity detected in the medium was very low.

### MMP9 expression in cultured endothelial cells

**Figure 9:**
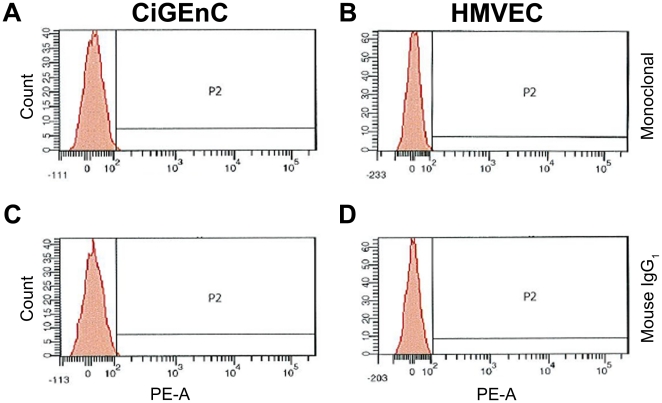
Lack of detectable MMP9 in the endothelial cells. MMP9 protein was not detected in either CiGEnC (panel A) or HMVEC (panel B) using mouse anti-human MMP9 antibody investigated by flow cytometry. The control antibody was negative (panels C and D). Results were reproduced in two separate experiments.

MMP9 has been shown to cleave VWF near the Tyr1606-Met1605 cleavage site for ADAMTS13 [9]. Using flow cytometry no MMP9 expression was detected either in CiGEnC or HMVEC cells (Figure 9A–9B). The control antibody did not exhibit any signal (Figure 9C–9D). As a positive control for antibody-binding 88% of the neutrophil population demonstrated labeling but no staining of the neutrophil population was observed with the control antibody (data not shown).

## Discussion

In this study we show that glomerular endothelial cells are capable of producing biologically active ADAMTS13, and that when ADAMTS13 is lacking, as in the ADAMTS13-deficient mouse, the glomerular capillary vessel wall is thickened and irregular. We presume that deposits of plasma proteins, such as VWF, contribute to platelet deposition along capillaries, as shown by electron microscopy. ADAMTS13, in plasma and on the surface of endothelial cells, will cleave ULVWF at the surface of endothelial cells and prevent the accumulation of platelets on the glomerular capillary endothelium.

ADAMTS13 cleaves ULVWF when it is bound to the endothelial cell [22]. The interaction between ADAMTS13 and ULVWF attains physiological importance in the setting of endothelial cell injury. ADAMTS13-deficiency in itself is not sufficient for the development of thrombocytopenia as shown in the ADAMTS13-deficient mouse in which platelet counts were normal and thrombi were not visualized in the kidneys [21]. Introducing the CASA/Rk background enhanced the susceptibility to develop TTP. These mice developed thrombocytopenia and VWF-rich thrombi in the kidney. The pathological lesion was more pronounced in the presence of endothelial injury induced by Shiga toxin [21]. The role of VWF was conclusively shown by generating ADAMTS13- and VWF-deficient mice [23] which were protected from Shiga toxin-induced injury. The ADAMT13-deficient mouse has a propensity to platelet deposition on the irregular surface of glomerular endothelial cells, as shown here, and would thus be more susceptible to develop thrombi and consumptive thrombocytopenia during endothelial injury than the wild-type mouse.

ADAMTS13 synthesis by endothelial cells has been previously shown using human umbilical vein endothelial cells, human umbilical artery endothelial cells [15], human aortic endothelial cells and an endothelium-derived cell-line (ECV304) [14]. Taken together with the results presented herein, showing ADAMTS13 production in microvascular endothelial cells, including glomerular endothelial cells, the production of ADAMTS13 by the endothelium may constitute a major source of ADAMTS13 present on vessel walls. Plasma ADAMTS13 may presumably be pooled from several sources including the stellate cells of the liver [13] as well as endothelial cells lining the vasculature [14], [15]. This may explain why patients with TTP respond to plasma therapy which replaces ADAMTS13 deficient in plasma [24], which in the normal population could originate from the endothelium. ADAMTS13 in the circulation cleaves VWF multimers and prevents the formation of thrombi on the vascular endothelium [25]. High shear stress in the glomerular capillaries may require an additional protective mechanism and we speculate that the synthesis and secretion of bioactive ADAMTS13 in the glomerular endothelium may contribute a local protective effect on the glomerular endothelial cell surface.

Previous studies have shown that ADAMTS13 was secreted from endothelial cells to the medium [14], [15] and that secreted protease was active [14]. Extracellular ADAMTS13 was demonstrated in the medium of glomerular endothelial cells in the current study. Bioactivity in the cell medium was, however, very low. Although there are differences between results obtained using in vitro cultured cells and in vivo physiological conditions, we assume, based on these results, that glomerular endothelial cells secrete active ADAMTS13 in vivo.

In addition to ADAMTS13, four other proteases, mainly secreted from leukocytes, are capable of cleaving VWF at or near the same peptide bond as ADAMTS13: elastase, proteinase 3, cathepsin G and MMP9 [9]. The first three proteases mentioned are serine proteases and would be inhibited by the protease inhibitor cocktail used in this study. MMP9 cleaves VWF at the M^1606^-V^1607^ bond [9]. It is, like ADAMTS13, a metalloproteinase and would not be inhibited by the cocktail inhibitor. For this reason we checked for MMP9 production in the CiGEnC or HMVEC. MMP9 could not be detected by flow cytometry. This result is supported by a previous study performed on murine glomerular endothelial cells which showed undetectable MMP9 mRNA levels and very low protease levels in unstimulated cells [26]. In addition, MMP9 would be inactivated by chemical denaturants such as urea [9] which was used in the dialysis step of the activity assay described here. Furthermore, we could show that the VWF-cleaving activity in CiGEnC or HMVEC was specifically related to ADAMTS13 as it was markedly decreased when ADAMTS13 was immunoadsorbed using a highly specific antibody [19]. Thus we conclude that the VWF cleavage detected in this study is mainly related to ADAMTS13 activity.

In summary, this study shows that human glomerular endothelial cells and microvascular endothelial cells produce bioactive ADAMTS13 which may both contribute to the pool of circulatory ADAMTS13 as well as prevent thrombotic events from occurring on the endothelial cell surface. The glomerular capillaries have a unique form of hemodynamics surrounding vessel lumina with high tensile forces. The phenotypic finding of platelet deposits along the vascular wall in the ADAMTS13-deficient mice suggests that ADAMTS13 prevents platelet deposition on ULVWF multimers even under basal conditions. High shear forces and endothelial damage would promote a prothrombic state in the glomerular vasculature. ADAMTS13 at the surface of the glomerular endothelial cell would be expected to exert a local protective effect which could be crucial at sites of endothelial cell damage.

## Methods

### Mouse tissue

Renal sections from ADAMTS13 wild-type mice, *Adamts13^+/+^* (n = 5), and ADAMTS13-deficient mice, *Adamts13^−/−^* (n = 5), were used. These mice have been previously described [21], [27]. Two mice in each group were generated on a mixed genetic background 129X1/SvJ and C57BL/6J [21] and the other three mice were a cross between C57BL/6J and CAST/Ei [27]. Animal studies were carried out with the approval of the animal ethics committee of the University of Michigan and University of Iowa. Paraffin-embedded mouse kidneys were prepared as previously described [28], [29].

### Immunohistochemistry for detection of ADAMTS13 in murine renal tissue

Immunohistochemistry was performed on murine renal tissue to determine ADAMTS13 expression according to a previously described method [19]. ADAMTS13 was detected using polyclonal chicken-415 anti-mouse ADAMTS13 IgY (directed against a sequence after the second CUB domain) at 1.25 µg/ml. Chicken IgY (Jackson Immuno Research, Suffolk, UK) was used as the negative control. The signal was detected using rabbit anti-chicken/turkey IgY: HRP (1:250, H+L, Invitrogen, San Francisco). Specificity of the secondary antibodies used was tested by omitting the primary antibodies. Slides were examined by light microscope (Axiostar Zeiss, mounted with an AxioCam MRc5 camera; Carl Zeiss, Göttingen, Germany). Positive signal stained brown.

### Scanning electron microscopy of mouse kidney

Paraffin-embedded murine renal sections were mounted onto glass slides as above. Slides were prepared as previously described [30] and cut using a diamond knife. Sections were mounted on to aluminum stubs and coated with a 30 nm layer of gold. Glomerular capillary wall thickness was assessed by quantifying capillary walls in 30 glomerular profiles from each mouse. Immunoelectron microscopy was performed as previously described [31]. Platelets were detected with polyclonal goat anti-human integrin-*β*3 antibody (Santa Cruz Biotechnology, Santa Cruz, CA) at 1:800, which showed cross-reactivity with mouse protein [32]. Normal goat IgG was used as the control antibody (Santa Cruz Biotechnology). Gold-conjugated rabbit anti-goat IgG (20 nm; BB International, Cardiff, UK) at 1:20 was used as the secondary antibody. Immunolabelled tissues were coated with a thin layer of carbon instead of gold prior to microscopy. Platelet deposits were quantified by counting the number of platelets/mm^2^ in 30 glomeruli/mouse. Tissues were scanned using a JEOL JSM-350 scanning electron microscope (JEOL Ltd., Tokyo, Japan) at an acceleration voltage of 5 kV and a working distance of 10 mm.

### Culture of endothelial cells

Conditionally immortalized human glomerular endothelial cells (CiGEnC) were cultured as described [33] and used at passage 24-36. Cells were grown in endothelial growth medium 2 - microvascular (EGM2-MV) supplemented with growth factors as supplied and 5% fetal bovine serum (all from Lonza, Walkersville, MD) and 1x penicillin/streptomycin (PAA Laboratories Gmbh, Pasching, Austria). Cells were grown to confluence at the permissive temperature of 33°C and then allowed to differentiate for at least 14 days at the non-permissive temperature of 37°C. Human dermal microvascular endothelial cells (HMVEC, Cell Application Inc, San Diego, CA) at passage 3–9 were grown according to the manufacturer's instructions in the same medium. As a negative control for ADAMTS13 synthesis, Chinese Hamster Ovary (CHO) cells were grown in D-MEM supplemented with 1x penicillin/streptomycin, 1x non-essential amino acids and 10% fetal bovine serum (all from PAA Laboratories, Pasching, Austria). Cells were cultured in culture flasks (TPP AG, Trasadingen, Switzerland) for collection of RNA, medium, lysates and for flow cytometry experiments, and on chamber slides (Nunc, Roskilde, Denmark) for immunofluorescence. For experiments designed to detect ADAMTS13 mRNA, protein and activity, cells were washed with Dulbecco's phosphate buffered saline (PAA Laboratories) and incubated for 24 hours with the respective medium as above, but without serum.

In certain experiments, designed to determine ADAMTS13 activity in cell medium, CiGEnC and HMVEC cells were stimulated with various agonists. After 24 hrs of incubation with serum free medium, cells were stimulated with phorbol-12-myristate-13-acetate (PMA, Sigma–Aldrich, St Louis, MO) at 500 nM for 1 hr or with histamine 100 µM (Sigma-Aldrich) [34] for 15 min, alternatively with estradiol 1 nM (Sigma-Aldrich) [35] for a further 24 hrs. As a positive control for cell stimulation VWF release was measured in cell media by ELISA as described [36]. Of the stimulants used, only histamine exhibited a 2-fold increase in VWF release.

Cell media were collected in 1x Complete EDTA-free protease inhibitor cocktail (Roche Diagnostics, Mannheim, Germany) and centrifuged to remove cell debris. Media were concentrated 100-fold using Amicon ultracentrifugal filter (Ultracel-50K, Millipore, Cork, Ireland). Cells were washed, detached by trypsinization, washed and resuspended in cell buffer (10 mM Tris, 150 mM NaCl, pH 7.4), supplemented with 1x Complete EDTA-free protease inhibitor cocktail, at 0.7−1×10^6^ cells/100 µl cell buffer. Cells were lysed by repeated freezing and thawing followed by sonication (Grants Instruments, Cambridge, Royston, UK) and centrifuged to remove the debris [20]. Media and lysates were frozen at −80°C until assayed.

### Real-time PCR for ADAMTS13 in endothelial cells

RNA was isolated using RNeasy mini kit (Qiagen Gmbh, Hilden, Germany) and reverse transcribed using TaqMan® Gold RT-PCR Kit (Applied Biosystems, Carlsbad, CA) as previously described [19]. ADAMTS13 gene transcripts were identified by real-time PCR (ABI prism 7000; Applied Biosystems) with a probe directed against exons 28–29 (translating into part of the second CUB domain; Applied Biosystems, assay Id Hs00260148_ml). Normal kidney RNA pooled from 14 individuals as well as normal liver RNA (BD Biosciences Clontech, Palo Alto, CA) were used as positive controls [20]. CHO cell RNA was used as the negative control [15] as well as a no-template control. 18S ribosomal RNA (Applied Biosystems, assay Id: Hs99999901_s1) was used to standardize the data.

### Flow cytometry for detection of ADAMTS13 and MMP9

ADAMTS13 detection within cells was carried out using flow cytometry. CiGEnC and HMVEC cells were washed with Hanks' balanced salt solution (HBSS) and detached with trypsin-EDTA (both from PAA Laboratories). 3×10^5^ cells/ml cells were fixed in 1% paraformaldehyde (Sigma–Aldrich) for 30 min at room temperature, washed with HBSS and centrifuged at 200 g for 5 min. The cell pellet was then incubated with A10 monoclonal antibody (mouse anti-human ADAMTS13 IgG_2b-κ_ against the disintegrin domain [13]) at 20 µg/ml or SU19 polyclonal rabbit IgG anti-human ADAMTS13 at 10 µg/ml (directed against a sequence in the CUB2 domain [37]) for 20 min. The antibodies were diluted with 0.3% Triton X-100 (ICN Biomedicals, Aurora, Ohio) in phosphate-buffered-saline (PBS, Medicago AB, Uppsala, Sweden) and the incubation steps were carried out at room temperature. Cells were then washed with HBSS and incubated with rabbit anti-mouse IgG:phycoerythrin (PE) at 1∶100 or swine anti-rabbit IgG:fluorescein isothiocyanate (FITC) at 1:40 (both from Dako, Glostrup, Denmark) for 20 min. Normal mouse IgG_2b-κ_ (Abcam, Cambridge, UK) and normal rabbit IgG (Antibody AB, Lund, Sweden) were used as the negative control antibodies.

MMP9 expression was studied using mouse anti-human MMP9 (Santa Cruz Biotechnology, Santa Cruz, CA) at 10 µg/ml diluted with 0.3% Triton X-100 in PBS. Mouse IgG_1_ was the isotype control and rabbit anti-mouse IgG:PE (both from Dako) was the secondary antibody. Human neutrophils, which produce MMP9 [38], were used as positive control cells. Neutrophils were isolated from a healthy donor using a one-step density gradient centrifugation with Polymorphprep® (Nycomed, Oslo, Norway) as previously described [39].

Antibody binding was analyzed using BD FACSCanto™ II and FACS Diva software (Becton Dickson Immunocytometry Systems, San Jose, CA). Cells were identified on the basis of the forward scatter (FSC) and side scatter (SSC) profile. By setting a gate around the cells, 1000 events for endothelial cells and 5000 events for neutrophils were analyzed for PE or FITC fluorescence. Background fluorescence of the control antibodies was subtracted in all the experiments. Specificity of the secondary antibodies was tested by omission of the primary antibodies.

### Immunofluorescence for demonstration of ADAMTS13 in cells

Endothelial cells, grown on chamber slides, were washed and permeabilized with 0.3% Triton X-100 in PBS and ADAMTS13 expression was investigated using anti-ADAMTS13 antibodies: A10 at 5 µg/ml or SU19 at 10 µg/ml, as previously described [19]. The signal was visualized with goat anti-mouse IgG F(ab)_2_ FITC 1:20 (Dako) or goat anti-rabbit (H+L) F(ab)_2_ Alexa-Flour 488 1:150 (Molecular Probes, Eugene, OR). For the A10 antibody, normal mouse IgG_2b-κ_ was the isotype control antibody. In order to test the specificity of SU19, the antibody was preincubated with a 50-fold molar surplus of blocking peptide [20]. Specificity of the secondary antibodies was tested by omitting the primary antibodies. Cell nuclei were stained with 4′, 6-diamidino-2-phenylindole (DAPI) in the mounting medium (Vector Laboratories, Burlingame, CA).

### Immunoblotting for detection of ADAMTS13 in cell medium

ADAMTS13 expression in the cell media was investigated by immunoblotting. Cell medium from both endothelial cells, diluted 1:2 in sample buffer (0.01 mol/L Tris buffer, pH 6.8 containing 4% [w/v] sodium dodecyl sulfate [SDS], 8% glycerol [all from Sigma-Aldrich] and 1% bromophenol blue [LKB Products AB, Bromma, Sweden]) were subjected to sodium dodecyl sulfate-polyacrylamide gel electrophoresis (SDS-PAGE) followed by immunoblotting under non-reducing conditions, as previously described [20]. ADAMTS13 was detected with goat anti-human ADAMTS13 antibody, directed against residues 825-875 located in fourth Tsp1 (thrombospondin 1)-like domain (BL156, Bethyl Laboratories, Montgomery, TX, USA) at 2 µg/ml. Rabbit anti-goat IgG:HRP (Dako) at 1∶2000 was used as the secondary antibody. Purified recombinant ADAMTS13 [20], diluted 1:800 in the above-mentioned buffer was used as the positive control and serum free control medium was used as negative control. Signal was detected by chemiluminescence using ECL plus (Amersham Biosciences, Uppsala, Sweden).

### VWF multimer structure analysis

ADAMTS13 activity was analyzed by VWF multimer structure analysis as previously described [1], [19]. Cell lysates were activated with 10 mmol/l BaCl_2_ for 30 min at 37°C. VWF (L.F.B., Les Ulis, France) at 2 U/ml was added and the suspensions were dialyzed against 1.5 M urea, 5 mM Tris pH 8.3 (both from Sigma-Aldrich) overnight. Cell buffer was used as the negative control.

### Immunoblotting for identification of VWF cleavage products specific for ADAMTS13

Specific ADAMTS13 activity in lysates and media was further tested by detection of the 140 kDa and 176 kDa VWF fragments arising after cleavage at the 1605Tyr-1606Met residues in the A2 domain by SDS-PAGE [8]. Lysates and media were activated as described for multimer structure analysis and VWF was added. After dialysis, the samples were diluted 1:2 under reducing conditions with 2x SDS-PAGE sample buffer (0.125 M Tris, 20% glycerol, 4% SDS, 2% dithioerythritol, [all from Sigma-Aldrich] and 0.001% bromofenol blue [LKB products]). Immunoblotting was carried out using a combination of pooled mouse anti-human VWF antibody (specific for the 176 kDa fragment) at 1:500 and mouse M13 antibody anti-human VWF (specific for 140 kDa fragment) at 1∶500 (gifts from Z.Ruggeri, The Scripps Research Institute, La Jolla, CA) [8]. Goat anti-mouse IgG:HRP (Dako) at 1∶2000 was the secondary antibody and detection was carried out by chemiluminescence. Recombinant ADAMTS13 purified from cultured HEK293 cells transfected with the pIRESneo2 VCP HIS-vector encoding the human *ADAMTS13* gene [20] was used as the positive control at 1:50 dilution in cell buffer or control medium. VWF substrate diluted in cell buffer or cell medium subject to the same conditions was used as the negative control. Cell lysates and media were also run without the addition of VWF to ascertain that VWF cleavage products were solely from exogenously added VWF. To test for specificity of ADAMTS13 activity cell lysates were pre-incubated with 20 mM EDTA [40] to block metalloproteinase activity. In addition, ADAMTS13 was immunoadsorbed by incubation with a 100 molar surplus of SU19 anti-ADAMTS13 antibody followed by incubation with a protein-A sepharose (Amersham Biosciences, Buckinghamshire, UK) in order to remove the ADAMTS13-antibody complexes formed.

### Statistical analysis

Differences between ADAMTS13 wild-type and deficient mice with regard to vessel wall thickness and platelet deposits on capillary walls were assessed by the Mann-Whitney U-test. A *P*-value≤0.05 was considered significant. Statistical analysis was performed by GraphPad Prism software (GraphPad software, Version 5, La Jolla, CA).
